# *Flow Index* accurately identifies breaths with low or high inspiratory effort during pressure support ventilation

**DOI:** 10.1186/s13054-021-03855-4

**Published:** 2021-12-15

**Authors:** Filippo Albani, Federica Fusina, Gianni Ciabatti, Luigi Pisani, Valeria Lippolis, Maria Elena Franceschetti, Alessia Giovannini, Rossella di Mussi, Francesco Murgolo, Antonio Rosano, Salvatore Grasso, Giuseppe Natalini

**Affiliations:** 1 Department of Anesthesia and Intensive Care, Fondazione Poliambulanza, Brescia, Italy; 2 Department of Anesthesiology, Neurointensive Care Unit, Azienda Ospedaliera Universitaria Careggi, Florence, Italy; 3 Department of Anesthesia and Intensive Care, Miulli Regional Hospital, Acquaviva Delle Fonti, Bari, Italy; 4 Mahidol Oxford Clinical Research Unit (MORU), Bangkok, Thailand; 5 Department of Anesthesia and Intensive Care, Mater Dei Hospital, Bari, Italy; 6 Department of Emergency and Organ Transplantation, University of Bari, Bari, Italy

**Keywords:** Artificial respiration, Positive-pressure respiration, Intensive care units, Patient-ventilator interaction, Inspiratory effort

## Abstract

**Background:**

Flow Index, a numerical expression of the shape of the inspiratory flow-time waveform recorded during pressure support ventilation, is associated with patient inspiratory effort. The aim of this study was to assess the accuracy of Flow Index in detecting high or low inspiratory effort during pressure support ventilation and to establish cutoff values for the Flow index to identify these conditions. The secondary aim was to compare the performance of Flow index,of breathing pattern parameters and of airway occlusion pressure (*P*_0.1_) in detecting high or low inspiratory effort during pressure support ventilation.

**Methods:**

Data from 24 subjects was included in the analysis, accounting for a total of 702 breaths. Breaths with high inspiratory effort were defined by a pressure developed by inspiratory muscles (*P*_musc_) greater than 10 cmH_2_O while breaths with low inspiratory effort were defined by a *P*_musc_ lower than 5 cmH_2_O. The areas under the receiver operating characteristic curves of Flow Index and respiratory rate, tidal volume,respiratory rate over tidal volume and *P*_0.1_ were analyzed and compared to identify breaths with low or high inspiratory effort.

**Results:**

*P*_musc_, *P*_0.1_, Pressure Time Product and Flow Index differed between breaths with high, low and intermediate inspiratory effort, while RR, RR/*V*_T_ and *V*_T_/kg of IBW did not differ in a statistically significant way. A Flow index higher than 4.5 identified breaths with high inspiratory effort [AUC 0.89 (CI 95% 0.85–0.93)], a Flow Index lower than 2.6 identified breaths with low inspiratory effort [AUC 0.80 (CI 95% 0.76–0.83)].

**Conclusions:**

Flow Index is accurate in detecting high and low spontaneous inspiratory effort during pressure support ventilation.

**Supplementary Information:**

The online version contains supplementary material available at 10.1186/s13054-021-03855-4.

## Background

Inspiratory support should maintain the inspiratory effort into a physiological range, sustainable by the patient. Under-assistance can lead to excessive inspiratory effort, generating a potentially harmful transpulmonary pressure leading to regional lung stress [1] and myotrauma [2] whereas over-assistance is associated with diaphragmatic atrophy and dysfunction [3].

Nonetheless, assessing inspiratory effort during assisted mechanical ventilation remains a clinical challenge, since a validated and affordable method to quantitatively assess it at the bedside is not yet available [4]. Monitoring the esophageal pressure (*P*_es_), which is the gold standard to evaluate the pressure developed by the respiratory muscles (*P*_musc_), is relatively invasive and requires considerable technical expertise in order to correctly interpret the *P*_es_ waveforms [5, 6]. Breathing pattern parameters, such as tidal volume and respiratory rate, or the rapid shallow breathing index (RR/*V*_T_) [7] are used as surrogates to infer patient effort, but they may be inaccurate and misleading [8]. Other proposed measures, such as the airway occlusion pressure (*P*_0.1_) and the swing in airway pressure generated by respiratory muscle effort recorded during a brief airway opening occlusion at end-expiration (Δ*P*_occ_) are both affected by technical or conceptual limitations [9, 10]. In particular, *P*_0.1_ measured by mechanical ventilators has a measurement error of ± 2 cmH_2_O [10, 11]. Since the threshold for identifying excessive inspiratory effort is 3.5–4 cmH_2_O and 1 cmH_2_O for low inspiratory effort, this approximation is far from negligible. Moreover, in subjects with respiratory muscle weakness, *P*_0.1_ could be low even in the presence of a high effort and of insufficient inspiratory support. Concerning Δ*P*_occ_, it is useful only in evaluating high patient effort, it requires active intervention from the attending physician, it is not continuous and not all ventilators allow to perform occlusions during PSV [12, 13].

Recently, we demonstrated that the Flow Index, a numerical expression of the shape of the inspiratory flow-time waveform recorded during pressure support ventilation (PSV), is independently associated with patient effort [14]. The aim of the present study was to assess the accuracy of the Flow Index in detecting high or low inspiratory effort during PSV and to establish cutoff values for the Flow index to identify these conditions. As a secondary aim, we sought to compare the performance of the Flow index,of the breathing pattern parameters (RR, *V*_T_,and RR/*V*_T_) and of *P*_0.1_ in estimating inspiratory effort during PSV.

## Methods

This study analyzed data collected during the Flow Index study [14] and was approved by the local ethical committee (Comitato Etico della Provincia di Brescia, NP4622).

Data was collected in patients admitted to the Intensive Care Unit (ICU) of Fondazione Poliambulanza, Brescia, Italy, who met all of the following criteria: age > 18 years, dependence on invasive mechanical ventilation (i.e. not ready to wean or having failed a spontaneous breathing trial on the day of the study [15]), being in PSV, having an esophageal balloon catheter already in place. Exclusion criteria were: mean arterial pressure < 60 mmHg, systolic arterial pressure > 180 mmHg, heart rate < 40 min^−1^ or > 150 min^−1^, PaO_2_/FIO_2_ < 150 mmHg, pH < 7.35 with PaCO_2_ > 45 mmHg, diagnosis of head injury, intracranial hemorrhage or cerebral ischemia.

A detailed description of the study protocol and measurements, and of the Flow index derivation formulas, is available elsewhere [14]. The mechanical ventilators in use for the study were Maquet Servo-i (Solna, Sweden) and GE-Datex Ohmeda S/5 Engstrom (Helsinki, Finland).

### Protocol

In order to explore the whole spectrum of patient effort, three pressure support (PS) levels were applied to each patient: (1) the PS level at enrollment was defined as basal, (2) the lowest tolerated PS level without dyspnea while keeping the ratio between respiratory rate and tidal volume (RR/*V*_T_) < 100 min^−1^ L^−1^ was defined as low, and (3) the maximal tolerated PS level to achieve near relaxation was defined as high. The high PS level was obtained by progressively increasing the PS until all signs of inspiratory muscle activity disappeared after inspiratory triggering, as assessed by visual inspection of the waveform of *P*_es_, airway opening pressure (*P*_aw_) and airflow. In order to avoid lung injury, the peak airway pressure was limited to a maximum of 30 cmH_2_O, regardless of achieving complete absence of inspiratory muscle activity. The three levels of PS were randomly applied for 20 min each, and all the remaining ventilatory variables (FiO_2_, inspiratory trigger, expiratory trigger) remained constant throughout the study, as previously set by the attending physician.

### Measurements

At the end of each 20 min period of stable PS level, *P*_aw_ at the ventilator Y connector, *P*_es_, inspiratory and expiratory flow, *V*_T_ and RR were recorded for 5 min (Datex-Ohmeda S/5 Collect; Datex-Ohmeda Division, Instrumentarium Corp., Helsinki, Finland). The sampling rate was 100 Hz.

*P*_es_ was measured by an esophageal balloon catheter (Marquat Gbm, Boissy-St-Léger Cedex, France) connected to a pressure transducer (AS3/CS3; Datex-Engstrom Division, Instrumentarium Corp., Helsinki, Finland).

**Fig. 1:**
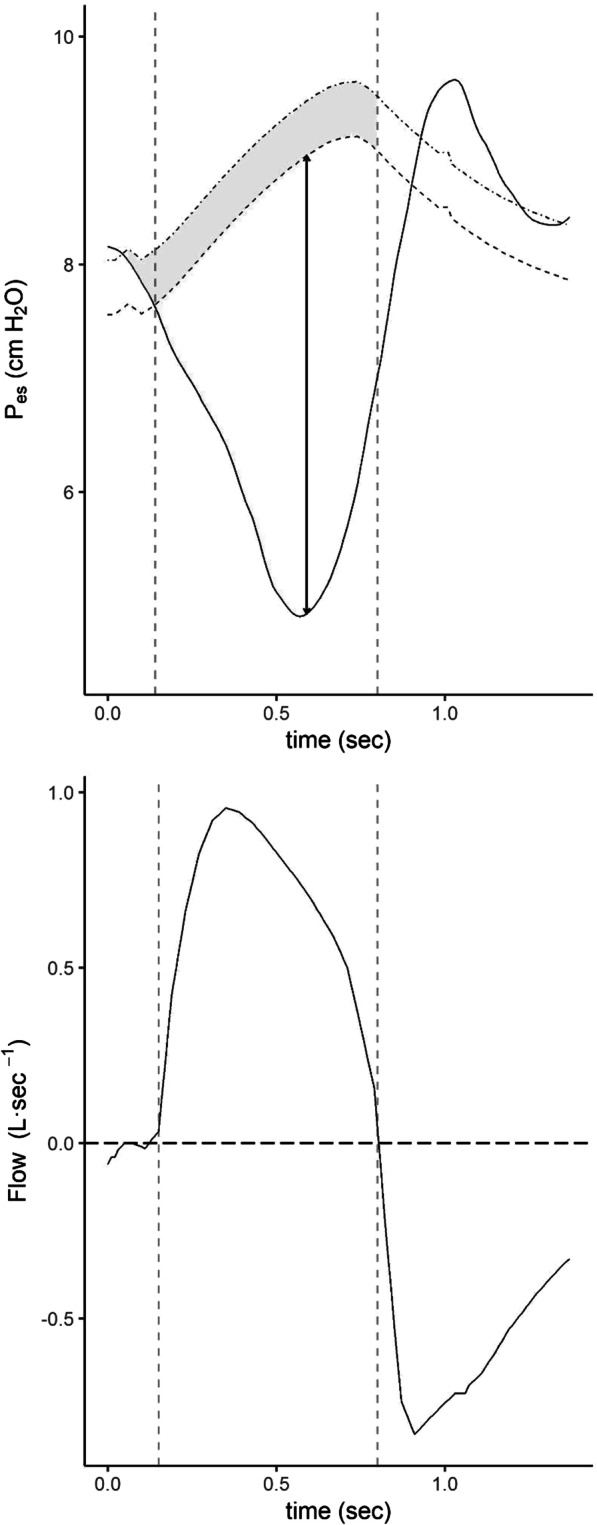
Airway pressure (*P*_aw_), esophageal pressure (*P*_es_) and airflow curves from a study participant. Upper panel: change in esophageal pressure (*P*_es_, continuous line) and in chest wall elastic recoil pressure (*P*_cw_, dashed line). The maximum pressure generated by respiratory muscles (*P*_musc_) is the maximal distance between *P*_cw_ and *P*_es_ (double arrow line). Lower panel: flow trace

The static recoil pressure of the chest wall (*P*_cw_) was calculated as the product of the *V*_T_ and the measured chest wall elastance (*E*_cw_). *E*_cw_ was obtained as the ratio between the inspiratory change in *P*_es_ [end-inspiratory plateau esophageal pressure (*P*_plat,es_) minus end-expiratory plateau esophageal pressure (*P*_exp,es_)] and *V*_T_ obtained during in a condition of near relaxation. The pressure generated by inspiratory muscles (*P*_musc_) was calculated as the maximal difference between *P*_cw_ and *P*_es_ (Fig. 1).

*P*_0.1_ was defined as the drop in esophageal pressure in the first 100 ms after the beginning of inspiration.

The Pressure–Time Product (PTP) was calculated as the area between *P*_cw_ and *P*_es_ during inspiratory flow multiplied by the respiratory rate, while PTP_tot_ was calculated by adding the area included between *P*_cw_ starting from the beginning of inspiratory effort and *P*_cw_ starting from beginning of inspiratory flow (grey area on Fig. 1) to PTP, multiplied by the respiratory rate. Lung compliance and resistance were also calculated using the Least Squares Fitting Method on transpulmonary pressure [16]. Respiratory system compliance and resistance were then calculated with the Least Squares Fitting Method on airway pressure [17] during low inspiratory effort (defined as *P*_0.1_ < 1.7 cmH2O [17] and PTP < 50 cmH2O s^−1^ min^−1^ [10]).

*P*_aw_, *P*_es_ and flow traces were independently reviewed by two authors (FA and GN) and patients were excluded if the data were not reliable or if evident artefacts were present. All consecutive breaths obtained from the longest portion of the esophageal pressure waveform without swallowing artifacts (detected by a transient, sudden increase on the pressure trace) were used for the analyses.

### The Flow Index

**Fig. 2:**
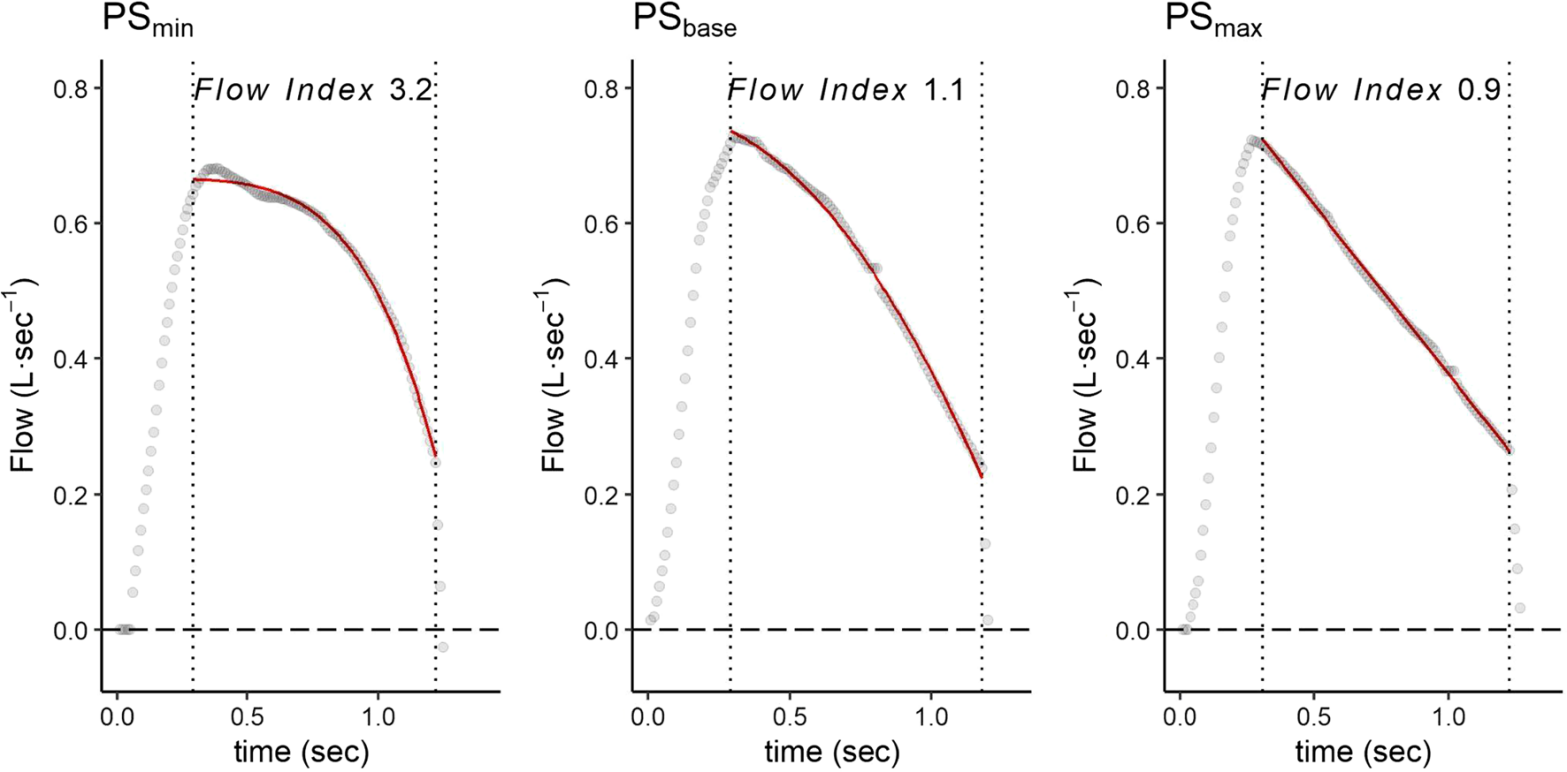
Procedure used to calculate *Flow Index* from the descending inspiratory portion of the flow waveform. Flow waveforms at the three different pressure support levels. The grey circles indicate the sampled inspiratory flow values, while the vertical lines indicate the cutting points used to select the descending inspiratory portion of the flow waveform. The red line shows the fitted model, calculated using Eq. 1 (detailed explanation in text). *Abbreviations* PS_min_, minimum pressure support; PS_base_, baseline pressure support; PS_max_, maximum pressure support

The detailed calculation of the Flow Index has been previously described [14]. Briefly, the portion of the inspiratory flow-time waveform included between the end of the ramp and before the expiratory trigger was fitted with the non-linear equation:

1 $$\dot{V} = a + b \cdot \Delta \,{\text{time}}^{{\text{c}}}$$

where the inspiratory flow ($\dot{V}$) is a function of time, of peak flow (a), of the rate of flow reduction (b) and of parameter c, which describes the downward facing concavity of the portion of the inspiratory flow waveform. The parameter c, calculated for every breath, was named *Flow Index*. The *Flow Index* describes the concavity of the curve using the same equation that computes the well-known *Stress Index*, which is calculated on airway pressure instead of inspiratory flow [18]. The *Flow Index* is equal to 1 when the inspiratory flow decreases linearly. If the waveform has an upward facing concavity, the *Flow Index* is < 1, whereas if the curve has a downward facing concavity, the *Flow Index* is > 1 (Fig. 2).

### Study outcome

The primary outcome was to validate the ability of the Flow Index in identifying low and high spontaneous inspiratory effort during PSV. In agreement with previously proposed cutoffs [6, 19, 20], breaths with high inspiratory effort were defined by a *P*_musc_ greater than 10 cmH_2_O while breaths with low inspiratory effort were defined by a *P*_musc_ lower than 5 cmH_2_O. As a secondary outcome, Flow Index was compared to other breathing pattern parameters used to monitor respiratory effort (RR, *V*_T_,and RR/*V*_T_) and to *P*_0.1_. In order to do so, we compared the areas under the receiver operating characteristic curves (AUC-ROC) of Flow Index and RR, *V*_T_, RR/*V*_T_ and *P*_0.1_ to identify breaths with low or high inspiratory effort.

### Statistical analysis

For the sample size calculation, we estimated a difference of 0.1 between the AUC of the Flow Index and the AUC of the RR/*V*_T_ in detecting breaths at low inspiratory effort. Considering a type one error rate set at 0.05, we calculated that 670 breaths would be necessary to obtain a power of 0.9, considering a ratio between cases and controls of 0.2 [21].

Continuous variables were described with mean (standard deviation) or median [1^st^-3rd quartile] and analyzed with the Student's *T*-test or Mann–Whitney test in accordance with their distribution. The categorical variables were expressed as counts (%) and analyzed with the Fisher exact test.

Logistic mixed effect models were used to obtain a probability of the occurrence of high or low effort while taking into account the aggregate structure of the data. Individual patients were entered as random-effects in these models and the fixed-effect used for each different model was the variable under analysis (Flow Index, RR, *V*_T_/kg of ideal body weight [IBW], RR/*V*_T_). After fitting the models, the conditional prediction was used to assess the AUC of the ROC curve and estimate sensibility, specificity, positive predictive value (PPV) and negative predictive value (NPV). Confidence intervals at 95% (CI 95%) were obtained with 2000 bootstrapped samples [22].

To evaluate the utility of other indices used in clinical practice, the same procedure was used to estimate the AUC of ROC curves of RR, *V*_T,_ RR/*V*_T_ and *P*_0.1_ and a bootstrap test was used to evaluate if the AUC of ROC curve of the Flow Index was statistically different.

To validate the obtained results, a stratified tenfold cross-validation was used and best thresholds, sensitivity, specificity, PPV and NPV were reported as mean (SD) for the tenfold obtained.

A sensitivity analysis with Pressure Time Product (PTP) as a grouping variable, both with and without threshold load (called PTP_tot_ and PTP, respectively), was also undertaken, using a value > 200 cmH_2_O s min^−1^ to identify high effort and a value of < 50cmH_2_O s min^−1^ to identify low effort [10].

The independent association between Flow Index and the respiratory system and lung mechanical properties (compliance and resistance) was evaluated using two linear mixed models, adjusting for *P*_musc_: Flow Index was the dependent variable, *P*_musc_ and resistance or compliance were explanatory variables.

All reported tests were 2-sided and a *P* value of less than 0.05 was considered significant. Statistical analyses were performed with R (R Core Team, 2020. R Foundation for Statistical Computing, Vienna, Austria) with packages “pROC” (version 1.17.0.1) and “lme4” (version 1.1-26) [23–25].

## Results

Twenty-eight patients were enrolled in the study. Four patients were excluded because the *P*_es_ trace was not reliable, therefore the data collected from 24 subjects was included in the analysis, accounting for a total of 702 breaths. Patients' characteristics on the day of enrollment and diagnosis at ICU admission are displayed in Table 1. Modification of PS resulted in a significant change in *P*_musc_, Flow Index, RR, RR/*V*_T_, *V*_T_/kg of IBW, *P*_0.1_, PTP and PTP_tot_, while minute ventilation did not change. As can be seen in Table 2, the levels of PS were different at high (*P*_musc_ > 10 cmH_2_O), intermediate (*P*_musc_ > 5 and < 10 cmH_2_O) and low effort (*P*_musc_ < 5 cmH_2_O).

**Table 1:** Patients’ characteristics

Age (years)	74 (10)
Female, *n* (%)	6 (25%)
Body Mass Index (kg m^−2^)	27 (7)
Height (cm)	168 (9)
Days on mechanical ventilation at enrollment	9 [3–21]
Patients with tracheostomy on study day, *n* (%)	7 (30%)
PEEP (cmH_2_O)	6 (1)
Expiratory trigger	0.25 [0.20–0.33]
Respiratory system compliance (mL cmH_2_O^−1^)	47 [35–80]
Respiratory system resistance (cmH_2_O L^−1^ s^−1^)	11 [7–15]
Lung compliance (mL cmH_2_O^−1^)	84 [37–129]
Lung resistance (cmH_2_O L^−1^ s^−1^)	12 [9–15]
FIO_2_	0.4 (0.08)
pH	7.46 (0.04)
PaCO_2_ (mmHg)	38 (5)
PaO_2_ (mmHg)	88 (25)
Hospital mortality, *n* (%)	4 (16%)
Total length of stay in ICU (days)	25 [15–35]
Main diagnosis at ICU admission	
Pneumonia	7 (29%)
COPD exacerbation	3 (12.5%)
Sepsis	4 (17%)
Trauma	3 (12.5%)
Other	7 (29%)

Data are shown as mean (standard deviation) or count (%) or median [1st–3rd quartile]. BMI, PEEP, respiratory system compliance and resistance, lung compliance and resistance, expiratory trigger and arterial blood gas analysis data were recorded at the time of patient enrollment

*PEEP* positive end expiratory pressure, *ICU* intensive care unit, *COPD* chronic obstructive pulmonary disease

**Table 2:** Ventilatory parameters at the three levels of PSV

	PS_low_	PS_base_	PS_high_	*P* value
*P*_musc_ (cmH_2_O)	6.2 (5.2–9.0) [2.4–20.5]	2.3 (1.8–6.7) [0.2–13.7]	1.3 (0.4–1.8) [0.1–11.8]	< 0.001
Flow Index	3.8 (2.7–5.4) [0.9–15.3]	2.6 (1.4–4.1) [0.7–11.0]	1.5 (1.1–1.9) [0.6–5.9]	< 0.001
RR (breaths min^−1^)	30 (23–35) [15–49]	25 (19–33) [12–47]	19 (16–25) [9–31]	0.001
RR/*V*_T_ (breaths⋅L^−1^ min^−1^)	74 (45–110) [27–135]	49 (31–78) [15–120]	28 (20–48) [7–69]	< 0.001
*V*_T_/IBW (mL kg^−1^)	6.9 (5.8–8.0) [3.4–10.4]	8.1 (6.7–9.4) [3.8–11.9]	10.6 (8.4–12.0) [5.9–16.1]	< 0.001
${\dot{\text{V}}}$E (L⋅min^−1^)	11.2 (8.3–13.6) [5.9–20.6]	11.2 (7.7–15.7) [5.0–20.1]	12.2 (8.8–13.8) [5.2–18.7]	0.979
*P*_0.1_ (cmH_2_O)	1.4 (0.9–1.8) [0.3–3.4]	1.0 (0.7–1.2) [0.1–2.4]	0.6 (0.2–1.0) [0.0–1.6]	< 0.001
PTP (cmH_2_O s min^−1^)	75.9 (65.3–150.6) [25.8–299.2]	25.4 (17.4–83.8) [1.1–201.1]	9.7 (2.0–12.8) [0.1–144.1]	< 0.001
PTP_tot_ (cmH_2_O s min^−1^)	197.01 (123.06–260.76) [33.5–437.0]	109.43 (39.84–179.63) [0.2–355.3]	35.05 (10.20–61.64) [2.6–257.8]	< 0.001
PS (cmH_2_O)	3 (1–4) [0–11]	8 (5–12) [1–14]	17 (14–20) [10–24]	< 0.001

Measurements for each subject were grouped by mean at the 3 different levels of pressure support. Data are shown as median (1st–3rd) [range] for the 24 subjects and P values obtained with Kruskal Wallis test at 3 different pressure support levels

*P*_musc_, pressure generated by respiratory muscles; RR, respiratory rate; *V*_T_, tidal volume; IBW, ideal body weight; ${\dot{\text{V}}}$E, minute ventilation; *P*_0.1_, airway occlusion pressure; PTP, pressure time product from the start of the inspiratory flow; PTP_tot_, pressure time product from the start of the inspiratory effort; PS, pressure support

It was possible to estimate respiratory system compliance and resistance in all but three patients [17], whereas lung compliance and resistance were calculated for all patients. Both lung and respiratory system mechanical properties were not independently associated with Flow Index (*P* values 0.91 and 0.36 for respiratory system compliance and resistance, and 0.20 and 0.09 for lung compliance and resistance, respectively).

*P*_musc_, *P*_0.1_, PTP, PTP_tot_ and Flow Index differed between breaths with high, low and intermediate inspiratory effort, while RR, RR/*V*_T_ and *V*_T_/kg of IBW did not differ in a statistically significant way (Table 3).

**Table 3:** Ventilatory parameters at high, intermediate and low inspiratory effort

	Low inspiratory effort	Intermediate inspiratory effort	High inspiratory effort	P
*P*_0.1_ (cmH_2_O)	0.7 (0.3–1.0)	1.2 (0.9–1.7)	1.6 (1.2–1.7)	< 0.001
*P*_musc_ (cmH_2_O)	1.6 (0.7–2.3)	6.3 (5.7–7.6)	12.9 (12.5–15.2)	< 0.001
Flow Index	1.6 (1.2–2.4)	3.4 (2.7–4.2)	8.1 (6.9–10.8)	< 0.001
RR (breaths min^−1^)	24 (16–30)	26 (20–34)	24 (22–26)	0.431
RR/*V*_T_ (breaths L^−1^ min^−1^)	48 (27–91)	56 (30–78)	33 (32–38)	0.421
${\dot{\text{V}}}$E (L min^−1^)	9.4 (7.3–13.5)	12.0 (9.9–15.4)	13.2 (11.7–15.4)	0.075
*V*_T_/IBW (mL kg^−1^)	8.1 (5.9–10.5)	7.9 (6.9–9.6)	10.0 (9.3–11.1)	0.124
PTP (cmH_2_O s min^−1^)	11.9 (3.6–22.7)	81.2 (73.9–108.1)	189.9 (159.9–205.3)	< 0.001
PTP_tot_ (cmH_2_O s min^−1^)	41.2 (19.0–79.4)	178.4 (121.9–209.9)	279.9 (243.9–344.6)	< 0.001
PS (cmH_2_O)	13 (8–17)	4 (3–6)	3 (1–10)	< 0.001

High inspiratory effort was defined as *P*_musc_ greater than 10 cmH_2_O. Low inspiratory effort was defined as *P*_musc_ lower than 5 cmH_2_O. P values were obtained with Kruskal Wallis test

*P*_0.1_, airway occlusion pressure; *P*_musc_, pressure generated by respiratory muscles; RR, respiratory rate; *V*_T_, tidal volume; IBW, ideal body weight; ${\dot{\text{V}}}$ E, minute ventilation; PTP, pressure time product from the start of the inspiratory flow; PTP_tot_, pressure time product from the start of the inspiratory effort; PS, pressure support

### Performance of Flow Index in detecting high inspiratory effort

**Fig. 3:**
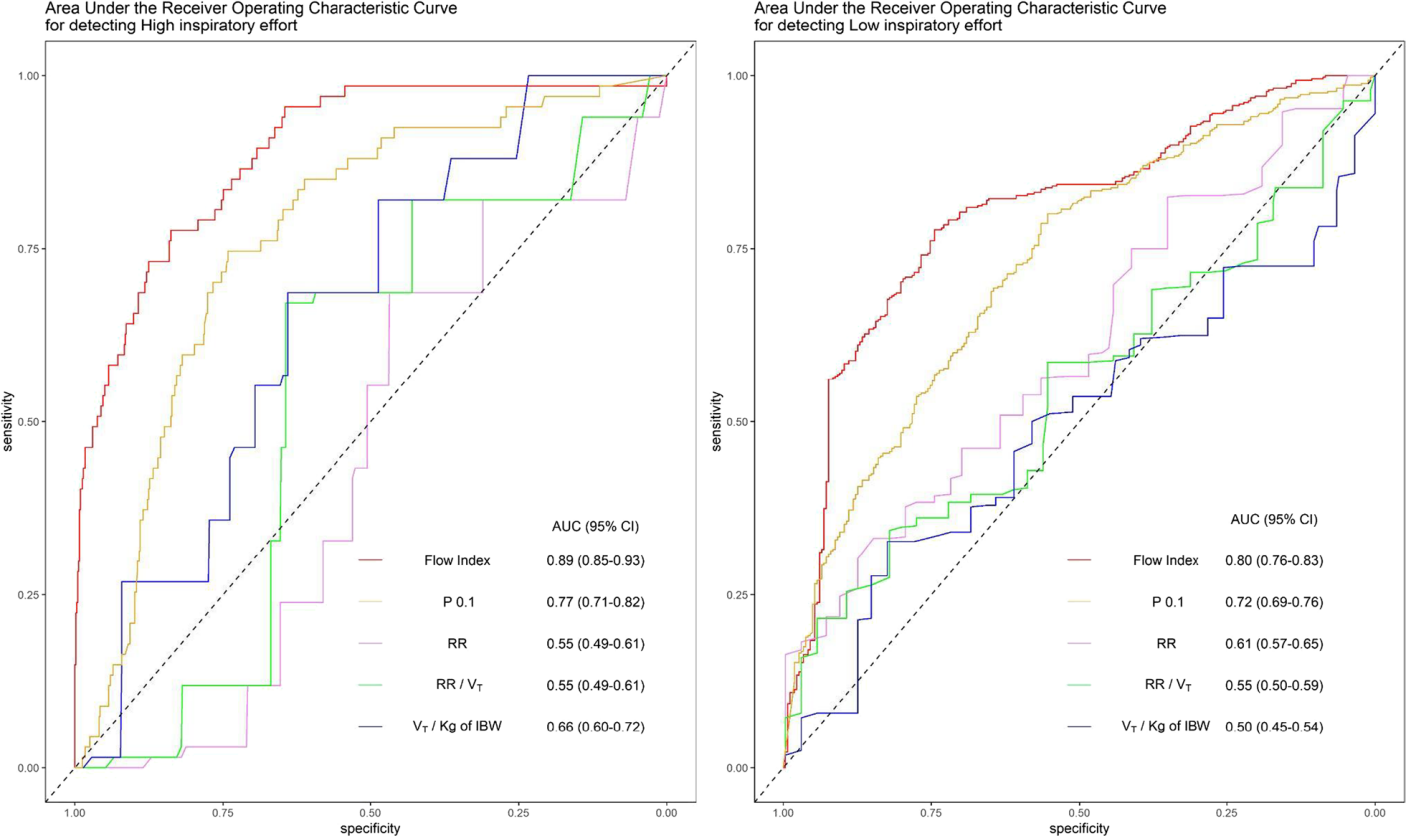
Areas under the receiver operating curve for detecting high (**A**) and low (**B**) inspiratory effort. Left panel: Areas under the receiver operating characteristic curves for detecting high inspiratory effort, defined as *P*_musc_ > 10 cmH_2_O. Flow Index is shown as a red curve, *P*_0.1_ as a yellow curve, respiratory rate as a pink curve, respiratory rate divided by tidal volume as a green curve, and tidal volume per kg of ideal body weight as a violet curve. Right panel: Receiver operating characteristic curve for detecting low inspiratory effort, defined as *P*_musc_ < 5 cmH_2_O. *Abbreviation* AUC, area under the receiver operating characteristic curve; CI, confidence interval; *P*_0.1_, airway occlusion pressure; RR, respiratory rate; *V*_T_, tidal volume; IBW, ideal body weight

Sixty-seven (10%) breaths were classified as breaths taken with high inspiratory effort. Respiratory variables during breaths taken at high inspiratory effort are displayed in Table 3. Flow Index was also significantly better at detecting high inspiratory effort compared to RR, RR/*V*_T_, *V*_T_/kg of IBW and *P*_0.1_ (*P* < 0.001 for all comparisons) as shown in Table 4 and Fig. 3A. The best threshold for the Flow Index was 4.5 (95% CI 3.0–5.1) with an AUC-ROC of 0.89 (95% CI 0.85–0.93) and an NPV of 0.98 (95% CI 0.97–0.99).

**Table 4:** Performance of the Flow Index and other routinely used parameters in etecting breaths with high inspiratory effort

	Threshold	Specificity	Sensitivity	PPV	NPV	AUC	*P* value
Flow index	4.5 (3.0–5.1)	0.84 (0.70–0.89)	0.81 (0.72–0.90)	0.34 (0.23–0.43)	0.98 (0.97–0.99)	0.89 (0.85–0.93)	–
RR (breaths min^−1^)	26 (25–26)	0.47 (0.58–0.51)	0.69 (0.58–0.79)	0.12 (0.10–0.14)	0.93 (0.91–0.96)	0.55 (0.49–0.61)	< 0.001
RR/*V*_T_ (breaths L^−1^ min^−1^)	40 (40–40)	0.64 (0.60–0.68)	0.67 (0.57–0.78)	0.17 (0.14–0.20)	0.95 (0.93–0.97)	0.55 (0.49–0.61)	< 0.001
*V*_T_/IBW (mL kg^−1^)	8.8 (8.8–8.8)	0.64 (0.59–0.69)	0.69 (0.58–0.81)	0.17 (0.14–0.19)	0.95 (0.94–0.97)	0.66 (0.60–0.72)	< 0.001
*P*_0.1_ (cmH_2_O)	1.4 (1.1–1.4)	0.75 (0.63–0.80)	0.75 (0.66–0.85)	0.24 (0.19–0.28)	0.97 (0.95–0.98)	0.77 (0.71–0.82)	< 0.001

Estimates of sensitivity, specificity, positive predictive value (PPV) and negative predictive value (NPV) for the best threshold (chosen by identifying the top-left corner value in the retrieving operating characteristic curve) for detecting breaths at high inspiratory effort using variables studied as predictors. 95% confidence intervals (95% CI) were obtained by 2000 bootstrapped samples. *P* values were computed by evaluating bootstrapping tests for the AUC of every variable versus the AUC of the Flow Index. *Abbreviations* RR, respiratory rate; *V*_T_, tidal volume; IBW, ideal body weight; *P*_0.1_, airway occlusion pressure; PPV, positive predictive value; NPV, negative predictive value; AUC, area under the retrieving operative characteristic curve

The internal tenfold cross validation yielded the same threshold for Flow Index, without significant modification in sensitivity, specificity, PPV and NPV (Additional file 1: Table S1).

### Performance of Flow Index in detecting low inspiratory effort

Four hundred and forty breaths (63%) recorded during the study protocol were classified as breaths with low inspiratory effort.

The AUC-ROC for Flow Index was 0.80 (95% CI 0.76–0.83), significantly greater than the AUC for RR, RR/*V*_T_, *V*_T_/kg of IBW *P*_0.1_ (Fig. 3B). A Flow Index lower than 2.6 (95% CI 2.2–2.9) predicted low inspiratory effort with a probability of 0.84 (95% CI 0.81–0.87), while a Flow Index greater than this threshold excluded low inspiratory effort with a probability of 0.66 (95% CI 0.61–0.71) (Table 5).

**Table 5:** Performance of the FI and other parameters in detecting breaths with low inspiratory effort

	Threshold	Specificity	Sensitivity	PPV	NPV	AUC	*P* value
Flow index	2.6 (2.2–2.9)	0.76 (0.70–0.82)	0.78 (0.70–0.82)	0.84 (0.81–0.87)	0.66 (0.61–0.71)	0.80 (0.76–0.83)	–
RR (breaths min^−1^)	24 (23–30)	0.63 (0.44–0.73)	0.53 (0.44–0.75)	0.70 (0.67–075)	0.44 (0.41–0.52)	0.61 (0.57–0.65)	< 0.001
RR/*V*_T_ (breaths L^−1^ min^−1^)	53 (53–53)	0.55 (0.50–0.62)	0.59 (0.54–0.63)	0.69 (0.66–0.72)	0.44 (0.41–0.48)	0.55 (0.50–0.59)	< 0.001
*V*_T_/IBW (mL kg^−1^)	8.1 (7.8–10.0)	0.58 (0.52–0.80)	0.50 (0.36–0.56)	0.67 (0.64–0.74)	0.41 (0.38–0.44)	0.50 (0.45–0.54)	< 0.001
*P*_0.1_ (cmH_2_O)	1.1 (0.9–1.3)	0.65 (0.56–0.73)	0.70 (0.61–0.80)	0.77 (0.74–0.80)	0.56 (0.51–0.64)	0.72 (0.69–0.76)	0.001

Estimates of sensitivity, specificity, positive predictive value (PPV) and Negative predicted value (NPV) for the best threshold (chosen with by top-left corner in the retrieving operative characteristic curve) for detecting breaths at low inspiratory effort using variables studied as predictors. 95% confidence intervals (95% CI) were obtained by 2000 bootstrapped samples. *P* values were computed by evaluating bootstrapping tests for the AUC of every variable versus the AUC of the Flow Index. *Abbreviation* RR, respiratory rate; *V*_T_, tidal volume; IBW, ideal body weight; *P*_0.1_, airway occlusion pressure; PPV, positive predictive value; NPV, negative predictive value; AUC, area under the retrieving operative characteristic curve

The tenfold cross validation confirmed this finding, estimating the same best threshold. Sensitivity, specificity, PPV and NPV were not altered (Additional file 1: Table S2).

Results of secondary analyses with PTP as grouping variables confirm the results of the primary analysis, except for the fact that Flow index and *P*_0.1_ have a similar diagnostic accuracy. (see Additional file 1: Tables S3, S4, S5 and Figures S1, S2).

Results of secondary analyses with PTP_tot_ as grouping variables show that *P*_0.1_ has a greater AUC-ROC than Flow Index. (see Additional file 1: Tables S6, S7, S8 and Figures S3, S4).

## Discussion

This study shows that the Flow Index is particularly accurate in identifying low inspiratory effort during PSV and in excluding high inspiratory effort, while it is less precise in discriminating against the occurrence of high inspiratory effort.

The rationale behind the Flow Index arises from the knowledge that the inspiratory flow is driven by the difference of pressure between airway opening and the alveoli. In the presence of a constant inspiratory pressure and in absence of patient effort, the flow is maximal at the beginning of inspiration and decreases exponentially, due to the decreasing pressure gradient between the airway opening and the alveoli, adopting an upward concavity.

On the other hand, in the presence of a sustained patient inspiratory effort, the fall in pleural pressure due to the patient's muscle activity decreases alveolar pressure promoting the instantaneous inspiratory flow. As a consequence, the shape of the flow waveform the active patient takes a downward concavity. The inspiratory waveform profile is quantified by the *Flow Index* whose value is proportional to the activation of the inspiratory muscles. In fact the equation used to calculate the Flow Index mirrors the one used to calculate the Stress Index on the airway pressure waveform [18]. We recently showed that the Flow Index correctly identifies the shape of inspiratory flow and that the Flow index value is proportional to the patient's inspiratory effort. Mechanical properties of the lung and respiratory system were not significantly associated with Flow Index in our analysis, strengthening the assumption that the shape of the portion of inspiratory flow analyzed by Flow index is dependent mainly on patient-ventilator interaction.

The inspiratory effort can have two components: one preceding the beginning of the inspiratory flow, which is not modified by the applied inspiratory pressure, and one following the beginning of the inspiratory flow, which is modified by the level of applied inspiratory support. Of note, the Flow Index is influenced by definition only by the inspiratory effort performed after the inspiratory trigger activation, and does not take into account the eventual threshold load due to auto PEEP and trigger sensitivity. Moreover, since the portion of the inspiratory flow-time waveform analyzed by Flow index is included between the end of the ramp and before the expiratory trigger, Flow Index is not influenced by the cycling-off criterion.

Having previously shown that the Flow Index is correlated to patient inspiratory effort on a breath to breath basis, the primary aim of the present study was to establish Flow Index’s cutoff values in order to identify breaths with low (i.e. *P*_musc_ lower than 5 cmH_2_O) or high (i.e. *P*_musc_ greater than 10 cmH_2_O) spontaneous breathing effort and thus identify the “normal” Flow index range in clinical practice. We found that the Flow Index accurately identifies low inspiratory effort when it is lower than or equal to 2.5 and, on the other hand, allows to rule out high inspiratory effort when it is lower than 4.5. As noted by its relatively low positive predictive value, a high effort could be absent in the presence of a Flow Index greater than 4.5. This could be explained by the fact that from a theoretical point of view the Flow Index is affected by the distribution of the total respiratory work between patient and ventilator [14]. Therefore, the Flow Index may be higher than 4,5 despite a *P*_musc_ of less than 10 cmH_2_O if the pressure applied by the ventilator is low, indicating that most of the work of breathing is performed by the patient. This notwithstanding, Flow Index still performed better in detecting high patient effort than all other analyzed indicators (i.e. RR, *V*_T_,RR/*V*_T_ and *P*_0.1_).

Our data show that the performance of RR, *V*_T_ and of RR/*V*_T_ in identifying high or low inspiratory effort during PSV is relatively poor, compared with the Flow Index. This is not surprising since, despite their widespread use in clinical practice in order to titrate PSV [8, 26–28], the performance of breathing pattern parameters has been seldom demonstrated except in physiological studies [29]. To our knowledge, only one clinically relevant study recently demonstrated that a relative bradypnea (i.e. less than 17 breaths/min) may accurately detect over-assistance [30].

In the past 25 years, several alternative methods to assess inspiratory effort have been proposed, namely the pressure muscle index (PMI), the least square fitting method, the inspiratory occlusion method (Δ*P*_occ_) and the airway occlusion pressure (*P*_0.1_) [9, 10, 20, 31, 32]. However, the PMI and least square fitting method did not show adequate accuracy in detecting the patient's inspiratory effort [20, 31, 32]. The Δ*P*_occ_, recently proposed to detect excessive inspiratory effort [9], cannot be continuously monitored and provides scarce information on the occurrence of low patient effort. *P*_0.1_ is an established measure to assess respiratory drive and is deemed as a surrogate to estimate patient effort [10]. Differently from the Flow Index, *P*_0.1_ evaluates the global inspiratory effort, both before and after the start of the inspiratory flow. While *P*_0.1_ gives better information on the neuro-ventilatory drive, the Flow index may be more suitable to assess the spontaneous inspiratory effort which is impacted by the changes in inspiratory support, since it explores the post-inspiratory trigger part of the inspiratory effort. In our cohort, when examining the inspiratory effort after the beginning of inspiratory flow on the single breath, Flow Index is a more robust indicator than *P*_0.1_. When evaluating effort over one minute (using PTP without threshold load), this difference is set aside, as predictable (the increase in respiratory rate increased PTP). Nonetheless, when including threshold load (using PTP_tot_), *P*_0.1_ is better than Flow Index in detecting abnormal effort. This makes us believe that *P*_0.1_ is better than Flow index in evaluating the total entity of inspiratory effort (including threshold load and respiratory rate), while Flow Index is better than *P*_0.1_ in evaluating inspiratory effort after the beginning of flow. We speculate that Flow Index might be more appropriate to assess the adequacy of the inspiratory support level (which acts only after the start of flow), while *P*_0.1_ might be more useful for a global assessment of the whole inspiratory effort, including threshold load. This hypothesis is in accordance with our data and with the physiological basis behind Flow Index and *P*_0.1_, but further studies are needed to confirm it and to assess the relationship between the information provided by these two indices, which likely should be deemed as complementary rather than alternative.

Other more recent techniques used to assess patient effort are diaphragm ultrasound [33] and continuous monitoring of the electrical activity of the diaphragm [34]. Yet, these are better suited for intermittent patient assessments and require a dedicated and costly catheter, respectively [35]. Moreover, the current data is insufficient to evaluate the diagnostic accuracy of both these methods for detecting high and low patient effort.

Flow Index has the potential to become a useful tool to assess patient effort in everyday clinical practice. Using a relatively simple software update, it could be implemented as a continuous measure to be visualized on the mechanical ventilator screen, similarly to the Stress Index [18, 36]. The bedside continuous availability at virtually no additional cost makes it a suitable marker for both high and low-resource settings.

This study has several limitations. It is a single center study, and its external validity needs to be assessed with further research in order for the results to be generalizable. Also, since the definitions of high and low patient inspiratory effort during PSV vary across the literature, different results might have been found for different cutoffs. Moreover, the Flow Index software has not yet been implemented on mechanical ventilators, therefore continuous bedside monitoring is not possible to date. In addition, the analyzed breaths were 702, with 9.8 analyzed breaths, on average, for each patient at each of the three PS levels. Even though this number of breaths is appropriate for the analysis conducted in this study (using the single breath as a statistical unit), it might not be sufficient to assess a stable patient inspiratory effort during a longer period of PSV. Further analyses are warranted in order to evaluate the performance of each patient’s mean Flow Index over a longer period of time, its impact on clinically meaningful outcome parameters and to validate the Flow Index as a clinical tool.

## Conclusion

Flow Index is accurate in detecting, continuously and non-invasively, high and low spontaneous inspiratory effort during PSV. These data support its potential application in clinical practice.

## Supplementary Information

**Additional file 1.** Supplementary material.

## Data Availability

The datasets during and/or analysed during the current study available from the corresponding author on reasonable request.

## Abbreviations

*P*_es_: Esophageal pressure
*P*_musc_: Pressure generated by respiratory muscles
RR: Respiratory rate
*V*_T_: Tidal volume
*P*_0.1_: Airway occlusion pressure
Δ*P*_occ_: Swing in airway pressure generated by respiratory muscle effort recorded during a brief airway opening occlusion at end-expiration
PSV: Pressure support ventilation
ICU: Intensive Care Unit
PS: Pressure support
*P*_aw_: Airway opening pressure
P_cw_: Static recoil pressure of the chest wall
*E*_cw_: Measured chest wall elastance
*P*_plat,es_: End-inspiratory plateau esophageal pressure
*P*_exp,es_: End-expiratory plateau esophageal pressure
AUC-ROC: Areas under the receiver operating characteristic curves
IBW: Ideal body weight
PPV: Positive predictive value
NPV: Negative predictive value
PTP: Pressure time product from the beginning of the inspiratory flow
PTP_tot_: Pressure time product from the beginning of the inspiratory effort
CI: Confidence interval
SD: Standard deviation
PEEP: Positive end expiratory pressure
PMI: Pressure muscle index
