# Supplementary material for: Flow Index accurately identifies breaths with low or high inspiratory effort during pressure support ventilation
Source: Crit Care. 2021 Dec 15;25:427. doi: 10.1186/s13054-021-03855-4 (PMC8672539; doi:10.1186/s13054-021-03855-4)
Supplement: Supplementary file 1 — Additional file 1. Supplementary material. [file 13054_2021_3855_MOESM1_ESM.docx]

**Supplementary material**

**Table 1S: 10-fold cross-validation. Performance of the Flow Index and other parameters in detecting breaths with high inspiratory effort.**

|  | **threshold** | **sensitivity** | **specificity** | **PPV** | **NPV** | **accuracy** |
| --- | --- | --- | --- | --- | --- | --- |
| **FI** | 4.53 (0.01) | 0.76 (0.15) | 0.84 (0.07) | 0.35 (0.15) | 0.97 (0.02) | 0.83 (0.07) |
| **RR** | 26 (1) | 0.38 (0.16) | 0.52 (0.08) | 0.08 (0.05) | 0.89 (0.02) | 0.51 (0.06) |
| **RR/V_T_** | 41 (0) | 0.68 (0.20) | 0.64 (0.06) | 0.16 (0.08) | 0.95 (0.04) | 0.65 (0.05) |
| **V_T_ IBW** | 8.8 (0.0) | 0.68 (0.18) | 0.64 (0.04) | 0.17 (0.07) | 0.95 (0.04) | 0.65 (0.04) |

Results of stratified 10-fold cross validation for the studied variable to internally validate the estimate of sensitivity, specificity, Positive Predictive Value (PPV) and Negative Predicted Value (NPV). The best threshold for detecting high inspiratory effort using variables studied as predictors was chosen by identifying the top-left corner value in the retrieving operating characteristic curve. Data are shown as mean (Standard Deviation).

Abbreviations: FI = Flow Index, RR = respiratory rate, V_T_ = tidal volume, IBW = ideal body weight, PPV = Positive Predictive Value, NPV = Negative Predictive Value, AUC = Area Under the retrieving operative characteristic Curve

**Table 2S: 10-fold cross-validation. Performance of the Flow Index and other parameters in detecting breaths with low inspiratory effort.**

|  | **threshold** | **sensitivity** | **specificity** | **PPV** | **NPV** | **accuracy** |
| --- | --- | --- | --- | --- | --- | --- |
| **FI** | 2.65 (0.00) | 0.77 (0.08) | 0.74 (0.07) | 0.83 (0.04) | 0.67 (0.08) | 0.76 (0.06) |
| **RR** | 24 (1.00) | 0.49 (0.08) | 0.61 (0.11) | 0.68 (0.09) | 0.42 (0.07) | 0.54 (0.07) |
| **RR/V_T_** | 53 (0) | 0.589 (0.05) | 0.56 (0.12) | 0.69 (0.10) | 0.44 (0.05) | 0.57 (0.06) |
| **V_T_ IBW** | 8.09 (0.00) | 0.50 (0.07) | 0.42 (0.09) | 0.59 (0.07) | 0.33 (0.08) | 0.47 (0.06) |

Results of stratified 10-fold cross validation for the studied variable to internally validate the estimate of sensitivity, specificity, Positive Predictive Value (PPV) and Negative Predicted Value (NPV). The best threshold for detecting low inspiratory effort using variables studied as predictors was chosen by identifying the top-left corner value in the retrieving operating characteristic curve. Data are shown as mean (Standard Deviation).

Abbreviations: FI = Flow Index, RR = respiratory rate, V_T_ = tidal volume, IBW = ideal body weight, PPV = Positive Predictive Value, NPV = Negative Predictive Value, AUC = Area Under the retrieving operative characteristic Curve

**Sensitivity analysis with Pressure Time Product from the beginning of inspiratory flow (PTP) as a grouping variable. Low inspiratory effort was defined as PTP < 50 cmH_2_O ‧ sec^-1^ ‧ min^-1^, high inspiratory effort as PTP > 200 cmH_2_O ‧ sec^-1^ ‧ min^-1^, intermediate inspiratory effort as 50>PTP< 200 cmH_2_O ‧ sec^-1^ ‧ min^-1^.**

**Table 3S: Ventilatory parameters in breaths with low, intermediate and high inspiratory effort**

|  | **Low inspiratory effort** | **Intermediate inspiratory effort** | **High inspiratory effort** | **p** |
| --- | --- | --- | --- | --- |
| **P_0.1_** (cmH_2_O) | 0.6 (0.3 to 1.0) | 1.2 (0.9 to 1.6) | 1.8 (1.4 to 2.3) | <0.001 |
| **P_musc_** (cmH_2_O) | 1.6 (0.7 to 2.1) | 6.4 (5.6 to 8.6) | 16.6 (15.2 to 18.6) | <0.001 |
| **Flow Index** | 1.6 (1.2 to 2.5) | 3.4 (2.6 to 5.5) | 10.6 (7.7 to 10.8) | <0.001 |
| **RR** (breaths⋅min^-1^) | 22 (16 to 29) | 27 (20 to 33) | 24 (24 to 27) | 0.142 |
| **RR/V_T_** (breaths⋅L^-1^⋅min^-1^) | 48 (26 to 73) | 52 (33 to 80) | 39 (35 to 50) | 0.659 |
| **V̇E** (L⋅min^-1^) | 9.4 (7.3 to 13.1) | 12.7 (10.3 to 15.9) | 13.2 (12.7 to 14.0) | 0.059 |
| **V_T_/IBW** (ml⋅Kg^-1^) | 8.2 (6.3 to 10.6) | 7.9 (6.9 to 9.7) | 9.6 (8.7 to 9.8) | 0.800 |
| **PTP** (cmH_2_O⋅sec⋅min^-1^) | 11.4 (3.1 to 20.2) | 85.8 (73.5 to 130.3) | 209.6 (205.3 to 254.4) | <0.001 |
| **PS** (cmH_2_O) | 13 (10 to 17) | 4 (2 to 6) | 3 (2 to 5) | <0.001 |

P values are obtained with Kruskal Wallis test for non-parametric distribution. Abbreviations: P_0.1_= airway occlusion pressure, P_musc_= pressure generated by respiratory muscles, RR = respiratory rate, V_T_ = tidal volume, IBW = ideal body weight, V̇E= minute ventilation, PTP= Pressure Time Product from the beginning of inspiratory flow, PS = pressure support

**Table 4S: Performance of the Flow Index and other routinely used parameters in detecting breaths with high inspiratory effort defined as PTP> 200 cmH_2_O‧sec^-1^‧min^-1^)**

|  | **threshold** | **specificity** | **sensitivity** | **PPV** | **NPV** | **AUC** | **P value** |
| --- | --- | --- | --- | --- | --- | --- | --- |
| **Flow index** | 4.5  (2.7-5.7) | 0.82 (0.61-0.89) | 0.80 (0.68-0.96) | 0.14 (0.08-0.22) | 0.99 (0.99-1) | 0.86 (0.80-0.92) | - |
| **RR**  breaths⋅min^-1^ | 30  (23-30) | 0.70 (0.40-0.73) | 0.60 (0.44-0.84) | 0.06 (0.04-0.09) | 0.98 (0.97-0.99) | 0.61 (0.50-0.71) | <0.001 |
| **RR/V_T_**  breaths⋅L^-1^⋅min^-1^ | 59  (31-59) | 0.58 (0.32-0.62) | 0.56 (0.40-0.80) | 0.05 (0.03-0.06) | 0.97 (0.96-0.99) | 0.52 (0.41-0.62) | <0.001 |
| **V_T_/IBW**  ml⋅Kg^-1^ | 7.7  (7.7-10.4) | 0.53 (0.25-0.57) | 0.56 (0.44-1) | 0.04 (0.03-0.06) | 0.97 (0.96-1) | 0.55 (0.48-0.62) | <0.001 |
| **P_0.1_**  cmH_2_O | 1.6  (1.4-1.7) | 0.83  (0.70-0.87) | 0.76  (0.60-0.88) | 0.14  (0.08-0.18) | 0.99  (0.98-0.99) | 0.77  (0.67-0.87) | 0.129 |

High inspiratory effort was defined as PTP> 200 cmH_2_O‧sec^-1^‧min^-1^. Estimates of sensitivity, specificity, positive predictive value (PPV), and negative predictive value (NPV) for the best threshold (chosen by identifying the top-left corner value in the retrieving operating characteristic curve) for detecting high inspiratory effort using variables studied as predictors. 95% confidence intervals (95% CI) were obtained by 2000 bootstrapped samples. P values were computed by evaluating bootstrapping tests for the AUC of every variable versus the AUC of the Flow Index.

Abbreviations: RR = respiratory rate, V_T_ = tidal Volume, IBW = ideal body weight, P_0.1_= airway occlusion pressure, PPV = Positive Predictive Value, NPV = Negative Predictive Value, AUC = Area Under the retrieving operative characteristic Curve

**Figure 1S. Areas under the receiver operating curve for detecting high inspiratory effort (PTP > 200 cmH_2_O‧sec^-1^‧min^-1^)**


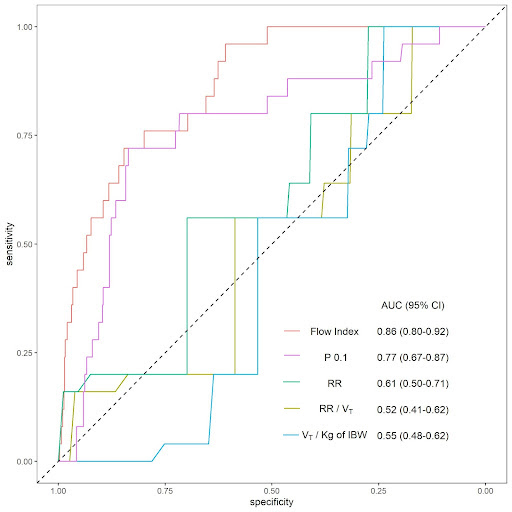


Abbreviations: P_0.1_= airway occlusion pressure, RR = respiratory rate, V_T_ = tidal volume, IBW = ideal body weight

**Table 5S: Performance of the FI and other parameters in detecting breaths with low inspiratory effort (defined as PTP<50 cmH_2_O‧sec^-1^‧min^-1^)**

|  | **threshold** | **specificity** | **sensitivity** | **PPV** | **NPV** | **AUC** | **P value** |
| --- | --- | --- | --- | --- | --- | --- | --- |
| **Flow index** | 2.5  (2.0-2.8) | 0.70 (0.63-0.79) | 0.73 (0.63-0.80) | 0.76 (0.73-0.81) | 0.66 (0.61-0.71) | 0.75 (0.71-0.78) | - |
| **RR**  breaths⋅min^-1^ | 25  (23-25) | 0.63 (0.57-0.71) | 0.59 (0.52-0.66) | 0.68 (0.64-0.72) | 0.54 (0.50-0.58) | 0.65 (0.61-0.69) | <0.001 |
| **RR/V_T_**  breaths⋅L^-1^⋅min^-1^ | 53  (33-53) | 0.56 (0.52-0.77) | 0.60 (0.44-0.66) | 0.65 (0.62-0.72) | 0.52 (0.48-0.56) | 0.59 (0.55-0.63) | <0.001 |
| **V_T_/IBW**  ml⋅Kg^-1^ | 8.1  (8.1-8.1) | 0.63 (0.58-0.69) | 0.54 (0.50-0.60) | 0.66 (0.62-0.70) | 0.51 (0.48-0.55) | 0.54 (0.50-0.59) | <0.001 |
| **P_0.1_**  cmH_2_O | 1.0  (0.9-1.1) | 0.67  (0.61-0.74) | 0.70  (0.63-0.76) | 0.74  (0.71-0.77) | 0.63  (0.58-0.67) | 0.73  (0.69-0.77) | 0.504 |

Low inspiratory effort was defined as PTP< 50 cmH_2_O‧sec^-1^‧min^-1^. Estimates of sensitivity, specificity, Positive Predictive Value (PPV), and Negative Predicted Value (NPV) for the best threshold (chosen with by top-left corner in the retrieving operative characteristic curve) for detecting low inspiratory effort using variables studied as predictors. 95% confidence intervals (95% CI) were obtained by 2000 bootstrapped samples. P values were computed by evaluating bootstrapping tests for the AUC of every variable versus the AUC of the Flow Index.

Abbreviations: RR = respiratory rate, V_T_ = tidal Volume, IBW = ideal body weight, P_0.1_= airway occlusion pressure, PPV = Positive Predictive Value, NPV = Negative Predictive Value, AUC = Area Under the retrieving operative characteristic Curve

**Figure 2S. Areas under the receiver operating curve for detecting low inspiratory effort (PTP< 50 cmH_2_O‧sec^-1^‧min^-1^)**

**
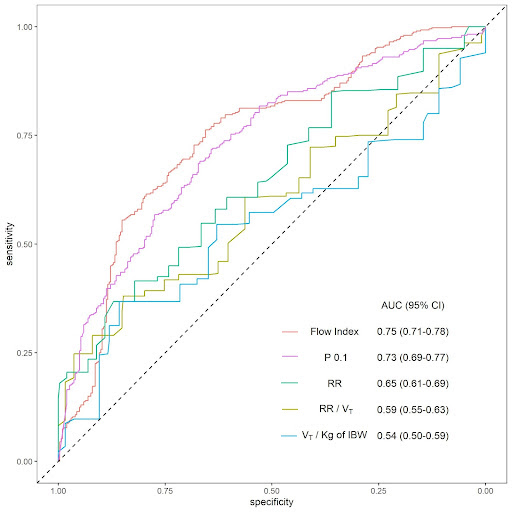
**

Abbreviations: P_0.1_= airway occlusion pressure, RR = respiratory rate, V_T_ = tidal volume, IBW = ideal body weight

**Sensitivity analysis with Pressure Time Product from beginning of inspiratory effort** (**PTP_tot_) as a grouping variable. Low inspiratory effort was defined as PTP_tot_ < 50 cmH_2_O ‧ sec^-1^ ‧ min^-1^, high inspiratory effort as PTP_tot_ > 200 cmH_2_O ‧ sec^-1^ ‧ min^-1^, intermediate inspiratory effort as 50>PTP_tot_< 200 cmH_2_O ‧ sec^-1^ ‧ min^-1^.**

**Table 6S: Ventilatory parameters in breaths with low, intermediate and high inspiratory effort**

|  | **Low inspiratory effort** | **Intermediate inspiratory effort** | **High inspiratory effort** | **p** |
| --- | --- | --- | --- | --- |
| **P_0.1_** (cmH_2_O) | 0.3 (0.2 to 0.7) | 0.9 (0.7 to 1.2) | 1.6 (1.3 to 2.0) | <0.001 |
| **P_musc_** (cmH_2_O) | 1.8 (1.1 to 2.3) | 4.8 (3.1 to 7.4) | 10.7 (6.6 to 13.3) | <0.001 |
| **Flow Index** | 1.5 (1.2 to 1.9) | 2.5 (1.6 to 3.7) | 5.2 (3.3 to 8.0) | <0.001 |
| **RR** (breaths⋅min^-1^) | 22 (15 to 27) | 24 (18 to 33) | 27 (23 to 30) | 0.057 |
| **RR/V_T_** (breaths⋅L^-1^⋅min^-1^) | 48 (30 to 66) | 50 (25 to 81) | 44 (32 to 90) | 0.841 |
| **V̇E** (L⋅min^-1^) | 8.7 (6.9 to 11.9) | 11.89 (9.5 to 14.3) | 14.2 (11.7 to 16.3) | 0.001 |
| **V_T_/IBW** (ml⋅Kg^-1^) | 8.2 (6.8 to 10.3) | 8.0 (6.2 to 9.9) | 9.6 (7.0 to 10.0) | 0.727 |
| **PTP_tot_** (cmH_2_O⋅sec⋅min^-1^) | 21.0 (4.9 to 30.4) | 112.5 (76.9 to 156.2) | 256.8 (228.7 to 351.9) | <0.001 |
| **PS** (cmH_2_O) | 21 (18 to 27) | 16 (11 to 20) | 12 (9 to 17) | <0.001 |

P values are obtained with Kruskal Wallis test for non-parametric distribution. Abbreviations: P_0.1_= airway occlusion pressure, P_musc_= pressure generated by respiratory muscles, RR = respiratory rate, V_T_ = tidal volume, IBW = ideal body weight, V̇E= minute ventilation, PTP_tot_= Pressure Time Product from beginning of inspiratory effort, PS = pressure support

**Table 7S: Performance of the Flow Index and other routinely used parameters in detecting breaths with high inspiratory effort (defined as PTP_tot_> 200 cmH_2_O‧sec^-1^‧min^-1^)**

|  | **threshold** | **specificity** | **sensitivity** | **PPV** | **NPV** | **AUC** | **P value** |
| --- | --- | --- | --- | --- | --- | --- | --- |
| **Flow index** | 2.9  (2.6-3.3) | 0.71 (0.66-0.76) | 0.78 (0.71-0.84) | 0.41 (0.37-0.46) | 0.92 (0.9-0.95) | 0.78 (0.73-0.82) | - |
| **RR**  breaths⋅min^-1^ | 25  (23-30) | 0.54 (0.45-0.72) | 0.62 (0.47-0.73) | 0.27 (0.24-0.32) | 0.85 (0.82-0.88) | 0.63 (0.58-0.68) | <0.001 |
| **RR/V_T_**  breaths⋅L^-1^⋅min^-1^ | 44  (44-46) | 0.61 (0.57-0.66) | 0.51 (0.43-0.6) | 0.26 (0.22-0.29) | 0.83 (0.8-0.85) | 0.55  (0.50-0.60) | <0.001 |
| **V_T_/IBW**  ml⋅Kg^-1^ | 8.8  (8.8-7.7) | 0.64 (0.5-0.68) | 0.51 (0.43-0.62) | 0.27 (0.23-0.31) | 0.83 (0.81-0.86) | 0.54 (0.49-0.59) | <0.001 |
| **P_0.1_**  cmH_2_O | 1.3  (1.3-1.3) | 0.78 (0.74-0.82) | 0.82 (0.76-0.88) | 0.50 (0.45-0.55) | 0.94 (0.93-0.96) | 0.85 (0.81-0.88) | 0.016 |

High inspiratory effort was defined as PTP_tot_> 200 cmH_2_O‧sec^-1^‧min^-1^. Estimates of sensitivity, specificity, positive predictive value (PPV), and negative predictive value (NPV) for the best threshold (chosen by identifying the top-left corner value in the retrieving operating characteristic curve) for detecting high inspiratory effort using variables studied as predictors. 95% confidence intervals (95% CI) were obtained by 2000 bootstrapped samples. P values were computed by evaluating bootstrapping tests for the AUC of every variable versus the AUC of the Flow Index.

Abbreviations: RR = respiratory rate, V_T_ = tidal Volume, IBW = ideal body weight, P_0.1_= airway occlusion pressure, PPV = Positive Predictive Value, NPV = Negative Predictive Value, AUC = Area Under the retrieving operative characteristic Curve

**Figure 3S. Areas under the receiver operating curve for detecting high inspiratory effort (PTP_tot_ >200 cmH_2_O‧sec^-1^‧min^-1^)**

**
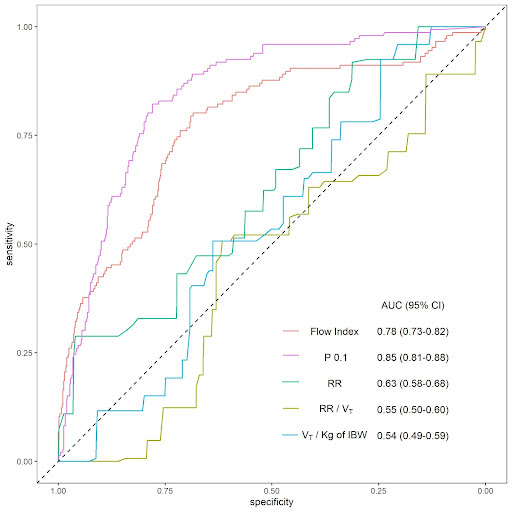
**

Abbreviations: P_0.1_= airway occlusion pressure, RR = respiratory rate, V_T_ = tidal volume, IBW = ideal body weight

**Table 8S: Performance of the FI and other parameters in detecting breaths with low inspiratory effort (defined as PTP_tot_<50 cmH_2_O‧sec^-1^‧min^-1^)**

|  | **threshold** | **specificity** | **sensitivity** | **PPV** | **NPV** | **AUC** | **P value** |
| --- | --- | --- | --- | --- | --- | --- | --- |
| **Flow index** | 2.0  (1.8-2.3) | 0.68 (0.6-0.73) | 0.73 (0.67-0.82) | 0.5 (0.46-0.54) | 0.85 (0.83-0.89) | 0.71 (0.67-0.75) | - |
| **RR**  breaths⋅min^-1^ | 25  (25-27) | 0.57 (0.5-0.62) | 0.75 (0.67-0.83) | 0.43 (0.40-0.46) | 0.84 (0.80-0.88) | 0.69 (0.65-0.73) | 0.543 |
| **RR/V_T_**  breaths⋅L^-1^⋅min^-1^ | 50  (37-70) | 0.53 (0.4-0.68) | 0.67 (0.51-0.88) | 0.38 (0.35-0.42) | 0.79 (0.75-0.89) | 0.62 (0.58-0.66) | <0.001 |
| **V_T_/IBW**  ml⋅Kg^-1^ | 8.2  (7.8-8.4) | 0.62 (0.55-0.68) | 0.58 (0.50-0.65) | 0.4 (0.36-0.45) | 0.77 (0.74-0.8) | 0.58 (0.53-0.62) | <0.001 |
| **P_0.1_**  cmH_2_O | 0.8  (0.6-0.9) | 0.71 (0.65-0.80) | 0.73 (0.65-0.80) | 0.52 (0.48-0.60) | 0.86 (0.83-0.89) | 0.79 (0.75-0.83) | <0.001 |

Low inspiratory effort was defined as PTP_tot_< 50 cmH_2_O‧sec^-1^‧min^-1^. Estimates of sensitivity, specificity, Positive Predictive Value (PPV), and Negative Predicted Value (NPV) for the best threshold (chosen with by top-left corner in the retrieving operative characteristic curve) for detecting low inspiratory effort using variables studied as predictors. 95% confidence intervals (95% CI) were obtained by 2000 bootstrapped samples. P values were computed by evaluating bootstrapping tests for the AUC of every variable versus the AUC of the Flow Index.

Abbreviations: RR = respiratory rate, V_T_ = tidal Volume, IBW = ideal body weight, P_0.1_= airway occlusion pressure, PPV = Positive Predictive Value, NPV = Negative Predictive Value, AUC = Area Under the retrieving operative characteristic Curve

**Figure 4S. Areas under the receiver operating curve for detecting low inspiratory effort (PTP_tot_< 50 cmH_2_O‧sec^-1^‧min^-1^)**


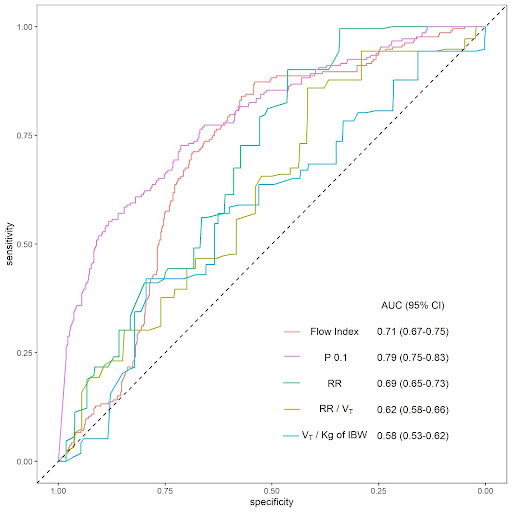


Abbreviations: P_0.1_= airway occlusion pressure, RR = respiratory rate, V_T_ = tidal volume, IBW = ideal body weight
